# Antox targeting AGE/RAGE cascades to restore submandibular gland viability in rat model of type 1 diabetes

**DOI:** 10.1038/s41598-024-68268-z

**Published:** 2024-08-06

**Authors:** Marwa M. Ahmad, Heba A. Hassan, Sara F. Saadawy, Enssaf Ahmad Ahmad, Naser Ahmed Mahmoud Elsawy, Manal Mohammad Morsy

**Affiliations:** 1 Department of Human Anatomy and Embryology, Faculty of Medicine, Zagazig University, Zagazig, Egypt; 2 Clinical Pharmacology Department, Faculty of Medicine, Zagazig University, Zagazig, 45519 Egypt; 3 Department of Pharmacology, Faculty of Medicine, Mutah University, Al-Karak, 61710 Jordan; 4 Medical Biochemistry Department, Faculty of Medicine, Zagazig University, Zagazig, Egypt

**Keywords:** Cell biology, Molecular biology, Structural biology

## Abstract

Diabetes mellitus (DM) is a chronic disorder of glucose metabolism that threatens several organs, including the submandibular (SMG) salivary glands. Antox (ANX) is a strong multivitamin with significant antioxidant benefits. The goal of this study was to demonstrate the beneficial roles of ANX supplementation in combination with insulin in alleviating diabetic SMG changes. For four weeks, 30 rats were divided into equal five groups (n = 6): (1) control group; (2) diabetic group (DM), with DM induced by streptozotocin (STZ) injection (50 mg/kg i.p.); (3) DM + ANX group: ANX was administrated (10 mg/kg/day/once daily/orally); (4) DM + insulin group: insulin was administrated 1U once/day/s.c.; and (5) DM + insulin + ANX group: co-administrated insulin. The addition of ANX to insulin in diabetic rats alleviated hyposalivation and histopathological alterations associated with diabetic rats. Remarkably, combined ANX and insulin exerted significant antioxidant effects, suppressing inflammatory and apoptotic pathways associated with increased salivary advanced glycation end-product (AGE) production and receptor for advanced glycation end-product expression (RAGE) activation in diabetic SMG tissues. Combined ANX and insulin administration in diabetic rats was more effective in alleviating SMG changes (functions and structures) than administration of insulin alone, exerting suppressive effects on AGE production and frustrating RAGE downstream pathways.

## Introduction

Salivary glands are the cornerstone of oral health. DM caused numerous insults to these glands and became one of the potential causes of hyposalivation in diabetic individuals^1^. The three paired major salivary glands are the parotid, submandibular, and sublingual glands. The SMG produce most of the salivary volume, or about 70% of daily secretions^2^.

Numerous studies have connected the development of diabetes to morphological changes in the salivary glands; according to Fouani^2^, the early onset of hyperglycaemia caused structural abnormalities in the intralobular duct. Furthermore, previous studies revealed substantial deterioration and shrinkage in the diabetic SMG, and vacuolisation confirmed their injury^3^.

Hyperglycaemia provokes oxidative stress through increased reactive oxygen species (ROS) generation as well as a reduction in the total antioxidant capacity. These changes lead to the oxidation of amino acid residues, subsequent changes in their structure and activity, and finally the loss of the biological functions of the cells^4^.

Additionally, hyperglycaemia leads to the generation of covalent crosslinks between proteins, various cell molecules, and sugar-producing products called advanced glycation end products (AGEs)^2^. AGEs induce ROS generation with dampening in antioxidant systems. Yet, some AGEs are induced by or under oxidative conditions. So, AGEs share a role in chronic stress conditions in diabetes^5^.

RAGE are cell surface receptors that bind to multiple ligands, as AGEs, leading to sustained cellular dysfunction and tissue damage^6^. RAGE activation causes up-regulation of nuclear factor kappa b (NFκB) together with inducing a state of oxidative stress^7^. Activation of RAGE by AGEs leads to degranulation of specific vesicles containing interleukin-8 (IL-8). IL-8 activates successive steps through its receptors, IL-8Rs, as it mediates cell apoptosis by elevating intracellular calcium (Ca2 +) with calcineurin activation and increasing the expression of Fas ligand (FasL). Expressed FasL induces activation of caspases alongside elevated Bcl-2-associated X-protein (BAX) levels causing cell death and apoptosis^8^. Numerous studies have linked structural alterations in the salivary glands to the development of diabetes.

Several studies^9–11^ stated that hyperglycaemia caused structural changes in the intralobular duct of the salivary glands. Also, the epithelial cells of the excretory ducts concurrently showed cytoplasmic vacuolisation, confirming their damage. Ageing, or age-related hypofunction of the salivary glands, is the cause of AGE accumulation, which damages cells. In oral tissues, the irreversible production of AGEs raises the risk of pulpitis and periodontitis^12^. Furthermore, the earlier study^12^ mentioned that AGEs have been implicated in the development and exacerbation of oral and general diseases, due to the production of low-intensity chronic inflammation. AGEs, regardless of where they originate, have an adverse cumulative effect on human tissues based on dose and duration. The measurement of salivary AGEs may represent a novel method for both the prognosis of suggested future treatment procedures and the identification of diseases exhibiting low-intensity inflammation.

A redox imbalance is crucial for the progression of many diabetic complications by the activation of multiple metabolic pathways associated with hyperglycaemia. These pathways include the polyol pathway, hexosamine pathway, protein kinase C (PKC) pathway, AGE pathway, and Rho-associated coiled-coil containing protein kinase (ROCK) pathway. PKC phosphorylates nicotinamide adenine dinucleotide phosphate (NADPH) oxidase subunits, which increases the production of ROS and can harm the kidneys and other organs^13^.

Through the PKC-dependent activation of NADPH oxidase and altered mitochondrial metabolism, high glucose levels cause cellular ROS. This, in turn, increases the production of AGEs, accelerated extracellular matrix (ECM) synthesis, and renal fibrosis^14^.

Ascorbic acid, Vitamin A acetate, and Vitamin E are among the multivitamins found in Antox (ANX), a powerful non-enzymatic antioxidant that also contains selenium. Its strong free radical-scavenging activity can protect against free radical attacks on mitochondrial membranes and mitochondrial DNA and thus protect against diabetic oxidative stress^15^.

Vitamins C and E and selenium each exhibited suppressing roles against AGE production, as reported by previous research^16–18^, respectively. Vitamin C, in addition, reduces apoptosis and necrosis in diabetic patients^19^. Vitamin E, too, has anti-inflammatory and anti-apoptotic capabilities^20^. Moreover, selenium has an anti-inflammatory effect by reducing the expression of the cytokine IL-8^21^.

Traditional antioxidant strategies in the treatment of diabetic complications are based on the use of either natural enzymes, such as SOD, catalase, or GPX, or antioxidant xenobiotics^22,23^. These usually act by donating an electron or a hydrogen atom from their phenolic or polyphenolic structures, hydroxyl groups, or thiol moieties to prevent the proliferation of ROS^24^. Antox is a mix of various antioxidants in a comprehensive formulation, potential synergistic effects, and a focused approach to oxidative stress management may provide significant advantages over other antioxidant techniques. ANX is not only an antioxidant but also an effective antioxidant that uniquely prevents the destruction of macromolecules caused by the excessive production of ROS^25^.

Due to the previously reported capabilities related to ANX as multivitamins, multivitamins promote submandibular gland health by supplying vital nutrients, antioxidants, and mechanisms to protect against oxidative stress and diabetes consequences. Incorporating multivitamin supplements into diabetic management techniques will help protect SMG integrity and function, resulting in better dental health outcomes for diabetics^26,27^.

The study aimed to assess the synergistic effects of ANX supplementation with insulin therapy in alleviating diabetic SMG alterations caused by STZ in a rat model with clarification of the possible mechanism related to AGE production and RAGE downstream cascades by analysing functional, biochemical, and histopathological data obtained from SMG.

## Material and methods

### Chemicals and animals

STZ with a CAS number of 18883-66-4 was purchased from Sigma Chemical Co. in Cairo, Egypt. ANX was purchased from a local pharmacy as ANTOX ® tablets from MEPACO-MEDIFOOD, Egypt. Insulin (Mixtard 30/70 vial) was purchased from Novo Nordisk, Denmark, consisting of 30% dissolved insulin and 70% isophane insulin (100 IU/1 ml) of human insulin. Insulin Mixtard 30/70 is a blend of intermediate- and short-acting insulin, administering it once daily offers a mix of basal and prandial insulin coverage^28^.

Thirty adult male Sprague–Dawley albino rats were used in this study, with an average weight of 250–300 g. The rats were purchased from the Experimental Animal House at the Faculty of Veterinary Medicine, Zagazig University, and kept in wire cages in the animal house of the Faculty of Medicine, Zagazig University, Egypt. After acclimatisation for two weeks, rats were divided into five groups of six rats each. The animals were maintained under steady environmental and nutritional conditions throughout the four-week duration of the experiment: temperature around 23 °C, humidity around 60%, 12-h light/dark cycles, and ad libitum access to food and water. All the experimental steps were carried out according to the International Guidelines for the Care and Use of Laboratory Animals and were ethically approved by the Institutional Animal Care and Use Committee (IACUC) of Zagazig University (ref. number; ZU- IACUC/3/F/203/2021).

### Induction of type 1 DM and administration of ANX

To induce type 1 DM experimentally, each rat received a single intraperitoneal (i.p.) dose of 50 mg/kg body weight of STZ dissolved in citrate buffer on the first day of the experiment^29^. On the second day, blood glucose levels were assessed by a glucometer using samples from the animals' tail veins. DM was verified at a fasting blood glucose level of 200 mg/dl or above.

Antox tablets (ANX) were crushed and dissolved in distilled water. A single daily dose of 10 mg/kg of ANX was administered from the first day of the experiment to its end^30^ in a volume that did not exceed 0.3 ml/100 gm through oral gavage using a polyethylene cannula 0.5 mm. Each ANX tablet contains selenium yeast (1000 mcg/gm) 55.7 mg (equivalent to selenium 55.7 mcg) + Vitamin A acetate (500 IU/mg) 4.67 mg (equivalent to Vitamin A 2036.46 IU) + ascorbic acid (Vitamin C) (97%) 92.78 mg (equivalent to ascorbic acid 100% 90 mg) + Vitamin E acetate (50%) 32.92 mg (equivalent to Vitamin E 15 mg = Vitamin E 50% 30 mg).

[Vitamin A (Retinol, Retinoic acid, Beta-Carotene), selenium, Vitamin C (Ascorbic Acid) and Vitamin E (Tocopherol)].

### Study design and animal

Rats were weighed and randomly allocated into five groups of six rats each:

1. *Control group*: Rats were kept without any treatment.
2. *DM group*: Rats received a single i.p. dose of 50 mg/kg of STZ dissolved in citrate buffer^29^.
3. *DM* + *ANX group*: Rats received a single i.p. dose of 50 mg/kg of STZ dissolved in citrate buffer and then administered ANX at a dose of 10 mg/kg/once daily/orally for four weeks. ANX dose selection was based on previous experiments that proved the desired protective effects against heavy metal damages in the brain of adult male albino rats were produced by ANX 10 mg/kg/day^31^, oxidative stress and cellular toxicity induced by sorafenib in male albino rats^30^, and reproductive toxicity induced by STZ in male rats^15^. ANX (10 mg/kg/day) proved protective against parotid gland insults induced by STZ in male rats^11^.
4. DM + insulin group
  Rats were injected with a single i.p. dose of 50 mg/kg of STZ dissolved in citrate buffer and then injected daily with a single SC dose of 1 U/100 g of insulin for four weeks^32^.
5. DM + insulin + ANX group
  Rats received a single i.p. dose of 50 mg/kg of STZ dissolved in citrate buffer and were then injected daily with a single s.c. dose of 1 U/100 gm insulin in addition to oral ANX administration, once daily in a dose of 10 mg/kg/day for four weeks.

### Weight of SMG and saliva flow rate

An intraperitoneal injection of pentobarbital (80 mg/kg) was used for anaesthetisation. Then, pilocarpine hydrochloride (2 mg/kg/i.p.) was administered to stimulate saliva secretion. The rat was placed in a vertical position (head-down). Pre-weighed cotton balls were used to collect saliva over 15 min and were weighed promptly on an electronic balance to avoid moisture loss^33^. The difference in pre- and post-collection cotton ball mass (in grams) indicates the flow rate of saliva from the submandibular salivary gland; later, it was converted into millilitres to be expressed as µL/minute. The saliva produced (total amount to be expressed by µl) was collected over 40 min and kept on ice in plastic tubes. The samples were centrifuged at 1540×*g* at 4 °C for five minutes. The supernatants were stored at − 80 °C for later analyses.

Blood samples were collected from the heart, stored in tubes containing EDTA (15%), and centrifuged at 1540×*g* for ten minutes, and the sera were refrigerated until analysis.

Finally, the rats were euthanised by isoflurane inhalation, and bilateral SMGs were removed and weighed. The right SMG was frozen and stored at − 80 °C for ribonucleic acid (RNA) extraction, while the left SMG was processed for histological analysis. The animal’s euthanasia was done under anaesthesia, inhaled isoflurane, and confirmed by cervical dislocation.

### Homogenization

Tissues were homogenized (PCV Kinematica Status Homogenator) in ice-cold phosphate-buffered saline (pH 7.4). The homogenate was sonified with an ultrasonifier (Branson 450 Sonifier) by three cycles (20-s sonications and a 40-s pause on ice). The homogenate was centrifuged (15,000×*g*, 10 min, 4 °C) and the cell-free supernatant was subjected to enzyme assay immediately^34^.

### Biochemical analysis

#### Estimation of fasting blood glucose (FBG) and HbA1c concentrations

At the end of the study, FBG and haemoglobin A1c (HbA1c) concentrations were estimated using a glucose-oxidase enzymatic commercial kit (Spinreact SAU, Sant Esteve de Bas, Spain) and the Colorimetric Spectrophotometry method (Crystal Chem, USA), respectively.

#### The salivary amylase alpha (α) enzyme concentration

Salivary amylase α content in saliva was detected by a rat salivary amylase Enzyme Linked-Immuno-Sorbent Assay (ELISA) kit (My BioSource, CA, USA) catalogue no: (#MBS3808889). The salivary amylase α1 ELISA Kit is intended for laboratory use only and is not for use in diagnostic or therapeutic procedures. The stop solution changes the colour from blue to yellow, and the intensity of the colour is measured at 450 nm using a spectrophotometer. To measure the concentration of salivary amylase α in the sample, this salivary amylase α ELISA Kit includes a set of calibration standards. The calibration standards are assayed at the same time as the samples and allow the operator to produce a standard curve of optical density versus salivary amylase α1concentration. The concentrations of salivary amylase α1in the samples were then determined by comparing the optical density (OD) of the samples to the standard.

#### Oxidative stress marker evaluation of SMG

The submandibular gland homogenate was created, aliquoted, and kept at − 80 °C until needed. The superoxide dismutase (SOD) and glutathione peroxidase (GPx) activities of each sample aliquot were estimated using spectrophotometric kits (Biodiagnostic, Giza, Egypt). SOD (Catalogue No: SD 25 21), GPx (Catalogue No: SD 25 11), and lipid peroxidation were measured by malondialdehyde (MDA) concentration using the thiobarbituric acid-reactive substances assay and absorbance was read at 532 nm^35^ (Catalogue No: SD 25 29).

#### Measurement of AGEs and proinflammatory parameter IL1β concentrations

Measurement of salivary AGE lysates was assessed with a competitive sandwich ELISA according to the manufacturing guidelines kit (My BioSource, CA, USA) catalogue no: (#MBS760916). This assay employs the competitive inhibition enzyme immunoassay technique. A monoclonal antibody specific to AGE has been pre-coated onto a microplate. A competitive inhibition reaction is launched between biotin-labelled AGE and unlabelled AGE (standards or samples) with the pre-coated antibody specific to AGE. After incubation, the unbound conjugate is washed off. Next, avidin conjugated to.

Horseradish Peroxidase (HRP) is added to each microplate well and incubated. The amount of bound HRP conjugate is proportional to the concentration of AGE in the sample. After the addition of the substrate solution, the intensity of the colour developed is inversely proportional to the concentration of AGE in the sample.

According to the manufacturer’s instructions, the IL1β concentration was measured in SMG tissue lysates using a rat IL-1 beta ELISA Kit (ab100768). This assay employs an antibody specific for rat IL-1 beta coated on a well plate. Standards and samples are pipetted into the wells and IL-1 beta present in a sample is bound to the wells by the immobilised antibody. The wells are washed, and a biotinylated anti-rat IL-1 beta antibody is added. After washing away unbound biotinylated antibodies, HRP-conjugated streptavidin is pipetted into the wells. The wells are again washed, a TMB (tetramethylbenzidine) substrate solution is added to the wells and colour develops in proportion to the amount of IL-1 beta bound. The stop solution changes the colour from blue to yellow, and the intensity of the colour is measured at 450 nm.

#### Protein measurement

Specific enzyme activities and oxidative protein damage were all related to protein concentrations, which were estimated by the Bradford method^36^ using bovine serum albumin as a standard.

#### Total RNA extraction and real-time quantitative RT-PCR (qRT-PCR) analysis

A quantitative real-time reverse transcription-polymerase chain reaction (RT-qPCR) was carried out to detect the expression levels of selected genes. The TRIZOL reagent (Invitrogen; Thermo Fisher Scientific, Inc., Waltham, MA, USA) was used to extract RNA from samples. Nanodrop (NanoDrop ND-1000 spectrophotometer; Thermo Fisher Scientific, Waltham, MA) was used to calculate the purity and integrity of RNA samples. Following the manufacturer's instructions, cDNA synthesis was carried out using a high-capacity cDNA Reverse Transcription Kit cDNA Kit; Applied Biosystems, USA) in a total volume of 20 μL as follows: A total of 10 μl containing 1 μg of RNA sample was added to 10 μL of 2X reverse transcriptase (RT) master mix. The resulting cDNA was stored at − 20 °C until used in the following PCR. The real-time RT-PCR was performed in an Mx3005P Real-Time PCR System (Agilent Stratagene, USA) using TOPreal™ qPCR 2X PreMIX (SYBR Green with low ROX) (Cat. # P725 or P750) (Enzynomics, Korea) by the manufacturer’s instructions using 10 µl TOPreal™ qPCR 2X PreMIX (SYBR Green with low ROX), 1 µl of each primer (forward and reverse), 5 µl cDNA template, and H_2_O PCR grade up to 20 µl with cycling conditions included a preparatory ten-minute denaturation at 95 °C followed by 40 cycles of denaturation at 95 °C for 20 s, annealing at 60 °C for 30 s, and extension at 72 °C for 30 s. The oligonucleotide-specific primers were synthesised by Sangon Biotech (Beijing, China) as demonstrated in Table 1. RNA expression of known housekeeping genes (glyceraldehyde-3-phosphate dehydrogenases GAPDH) was used to normalise the level of expression of the target genes. The 2^−ΔΔCt^ method was used to analyse the expression of each amplicon^37^.

**Table 1:** Primer sequences used for quantitative real-time PCR assay.

	Primer	Accession no
IL-1β	Forward (5′–3′ ): CACCTCTCAAGCAGAGCACAG Reverse (5′–3′ ): GGGTTCCATGGTGAAGTCAAC	M98820, NW_047658
IL8	Forward (5′–3′ ): CATTAATATTTAACGATGTGGATGCGTTTCA Reverse (5′–3′ ): GCCTACCATCTTTAAACTGCACAAT	NM_030845.1
TGF-β1	Forward (5′–3′ ): GGAGCCACTGCCCATCGTCTACTAC Reverse (5′–3′ ): GGAGCGCACGATCATGTTGGAC	NM_021578.2
TNFα	Forward (5′–3′ ): ACTGAACTTCGGGGTGATCG Reverse (5′–3′ ): GCTTGGTGGTTTGCTACGAC	NM_012675.3
NF-Κb	Forward (5′–3′ ): CAGGACCAGGAACAGTTCGAA Reverse (5′–3′ ): CCAGGTTCTGGAAGCTATGGAT	NM_199267.2
RAGE	Forward (5′–3′ ): TGCCCAGCCTCCCCCTCAAA Reverse (5′–3′ ): GGGTGGCCACGCAGCTGTAG	NM_053336.2
PKCβ	Forward (5′–3′ ): AGCCGGAGTGGATGGCTGGT Reverse (5′–3′ ): CTCGCTTTCTTCCGGCGGCA	M19007.1
GAPDH (Rat)	Forward (5′–3′ ): GGCACAGTCAAGGCTGAGAATG Reverse (5′–3′ ): ATGGTGGTGAAGACGCCAGTA	NM_017008.4

### Histological examination

The formalin-fixed sectioned left SMG specimens were processed for Hematoxylin and Eosin stain (H&E) and Masson’s Trichrome stain following Bancroft and Layton^38^. All stained sections were examined using light microscopy (LEICA DM500, Switzerland) and photographed with the attached five-mega-pixel Leica digital camera (ICC50 W, Switzerland) at the Image Analysis Unit of the Human Anatomy and Embryology Department, Faculty of Medicine, Zagazig University.

### Immuno-histochemical studies

For the immunohistochemistry detection of 8-hydroxy-2'-deoxyguanosine (8-OHdG), Alpha-smooth muscle actin (-SMA), and BAX, paraffin blocks of formalin-fixed SMG tissue specimens were used using the streptavidin–biotin immunoperoxidase technique (Dako- Cytomation, Glostrup, Denmark). Sections of 3–5 mm thickness were cut from formalin-fixed, paraffin-embedded blocks, placed on positively charged slides, deparaffinised in xylene, and rehydrated in graded alcohol. A citrate buffer (pH 6.0) was used to boil the sections for 20 min. They were then washed in PBS (pH 7.3). Sections were treated with 3% hydrogen peroxide to block the endogenous peroxidase activity. Thereafter, the sections were incubated with the primary antibody. These antibodies included Glypican-3 (GPC-3): mouse monoclonal antibody, dilution 1:100; (CM396, A, B, Clone IG12, Biocare Medical LLC, Concord, USA); 8-Hydroxydeoxyguanosine(8-OHdG): monoclonal antibody, dilution 1:100 (Clone 15A3, SC-66036, Santa Cruz, California, USA), mouse monoclonal antibody to α-SMA 1/500 dilution (Clone 1A4, ab7817, Abcam) in a humid chamber overnight, then washed three times with PBS (Ramos-Vara et al., 2008). For BAX sections, they were first incubated with 1% pre-immune rabbit serum to decrease nonspecific staining and then with a monoclonal antibody against BAX protein (1:200 dilution; Dako, Carpinteria, California, USA)^39^.

Incubation with a secondary antibody and product visualisation were performed (Dako-Cytomation). For 8-OHDG, diaminobenzidine substrate (Research Genetics, Huntsville, Alabama, USA) was the chromogen, and for α-SMA 3,3'-diaminobenzidine (DAB) hydrogen peroxide was used as a chromogen to stain α-SMA bounded structures and localise the site of immune reactions. For BAX, 3-amino-9-ethyl carbazole (AEC) diaminobenzidine substrates were used as chromoge; finally, immune-stained sections were counterstained using Mayer's haematoxylin.

### Histomorphometric analysis

Five independent H&E and immune-stained fields at a magnification of 400 were randomly selected from five different submandibular gland slices from each animal in each group. Image j software version “1.50i/Java 1.6.0_24” analyser computer system (Wayne Rasband, NIH, Bethesda, Maryland, USA), http://imagej.net/ij/, was used to measure the following: acinar diameter (AD) [μm], acinar epithelial height (AEH) [μm], striated duct diameter (SDD) [μm], striated duct epithelial height (DEH) [μm], area percentage (%) of collagen fibres and α-smooth muscle actin, and OD of 8-OHDG and BAX.

### Statistical analysis

Statistical analysis was done using GraphPad Prism software version 9.0.1 (GraphPad Software, Inc. La Jolla, CA, USA), www.Graphpad.com The Kolmogorov–Smirnov test for normality and Bartlett's test for the homogeneity of variances were used. The Tukey method was used for multiple comparisons via ANOVA. Data were represented as mean ± standard error. Probability values < 0.05 were determined as significant results.

### Ethics approval and consent to participate

Experiments and animal handling were designed in line with the instructions of international ethical guidelines for the care and use of laboratory animals, and complied with the ARRIVE guidelines and approved by the Institutional Animal Care and Use Committee, Zagazig university (ZU-IACUC) with approval no: ZU-IACUC/3/F/203/2021.

### Study limitations

The study included a relatively small sample size of six rats per group. While this is frequent in animal studies, it can reduce the findings’ generalisability and statistical power. The four-week treatment period may be insufficient to capture long-term benefits or potential gradual improvements in SMG function and structure. ANX was supplied at a set dose (10 mg/kg/day) in the trial, with no dose–response relationships or alternative dosing regimens investigated. Different ANX delivery doses or frequencies could be investigated to determine optimal therapy techniques and potential dose-related effects. Another limitation of our study is that we focused on the absolute weight of the submandibular gland rather than its size relative to the body weight. The DM group showed variability in body weight for various reasons, so there may be an inconsistent correlation between body weight and SMG weight. This may be due to genetic, developmental, or environmental factors. In pathological conditions such as DM, the disease might disproportionately affect organ size compared to overall body weight. Normalising by body weight in such cases might obscure significant findings related to the disease's impact on the organ.

### Goals

Some of our goals will be to increase the sample size and diversity of experimental subjects to improve the generalisability and statistical power of the findings, clarify the molecular processes that underpin the therapeutic effects of ANX supplementation in diabetic SMG dysfunction, and apply our preclinical findings in clinical practice by undertaking human clinical trials to assess the efficacy, safety, and feasibility of ANX supplementation in diabetic patients with SMG dysfunction. These trials will yield vital information on real-world medicinal uses and patient outcomes and optimise dose regimens, treatment durations, and combination therapies to enhance the therapeutic advantages of ANX supplementation in diabetic SMG problems. Dose–response studies and pharmacokinetic analysis will help establish evidence-based treatment practices.

By addressing these limits and pursuing our aims, we hope to advance the field of diabetic SMG research, improve therapy outcomes, and eventually improve the quality of life for people suffering from this devastating consequence of DM.

## Results

### ANX plus insulin reversed low SMG weight in diabetic rats

**Figure 1:**
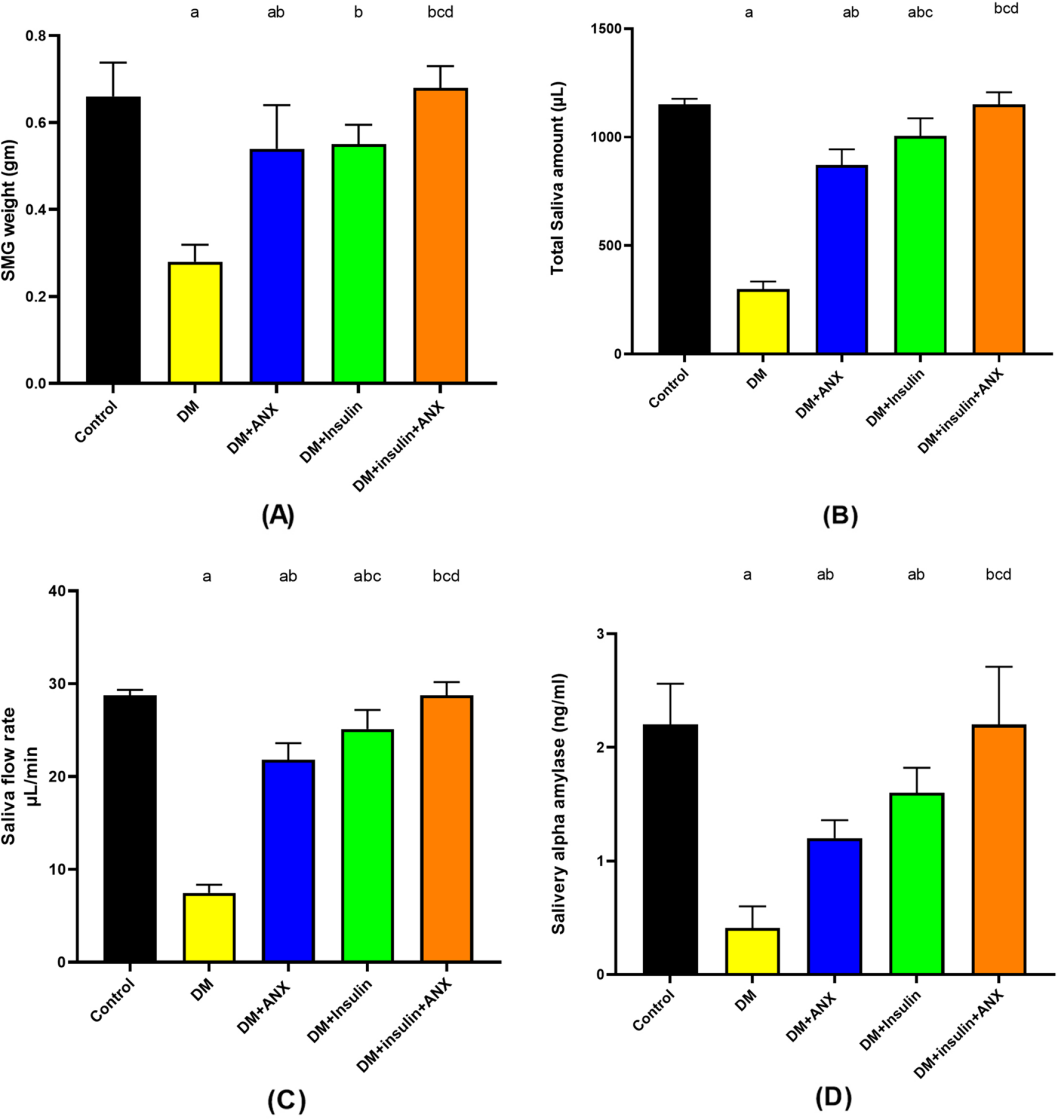
Effect of ANX plus insulin on SMG weights (**A**), total saliva amount (**B**), saliva flow rate (**C**) and salivary alpha-amylase enzyme (**B**) in STZ-induced diabetic rats.Data were presented as the mean ± SE (n = 6). ^a^ *p* < 0.05 versus control group; ^b^ *p* < 0.05 versus DM group; ^c^ *p* < 0.05 versus DM + ANX group and ^d^ *p* < 0.05 versus DM + insulin group (STZ, streptozotocin; ANX, antox; DM, diabetes mellitus and SMG, submandibular gland).

When compared to the control group, DM showed a statistically significant low SMG weight (*p* < 0.0001). The DM + ANX group showed increased SMG weight compared to the DM group (*p* < 0.0001) but not to the control group (*p* = 0.02). When compared to the DM + insulin group, SMG weight was significantly higher. The increase was more pronounced in the DM + insulin + ANX group when compared to the administration of the DM + ANX or DM + insulin groups (*p* = 0.01 and *p* = 0.02, respectively) Fig. 1A.

### ANX with insulin treatment elevated the decreased total amount of saliva, saliva flow rate, and salivary amylase α enzyme in diabetic rats

Analysis of the saliva of the DM group revealed a significant decrease in total saliva amount (Fig. 1B), saliva flow rate (Fig. 1C), and salivary amylase α (Fig. 1D) concentration compared to the other groups (*p* < 0.0001, each). In comparison to the DM group, both the DM + ANX and DM + insulin groups considerably increased the total amount of saliva, the flow rate of saliva, and the salivary amylase α concentration (*p* < 0.0001, *p* = 0.02, and *p* < 0.0001, respectively), though they remained below the normal level. However, the increases in the total amount of saliva, the flow rate of saliva, and the salivary amylase α concentration were more significant in the DM + insulin + ANX group than in the DM + ANX or DM + insulin groups (*p* < 0.0001, *p* < 0.0001, and *p* = 0.02, respectively).

### Biochemical results

#### FBG concentration and glycosylated haemoglobin (HbA1c)

**Figure 2:**
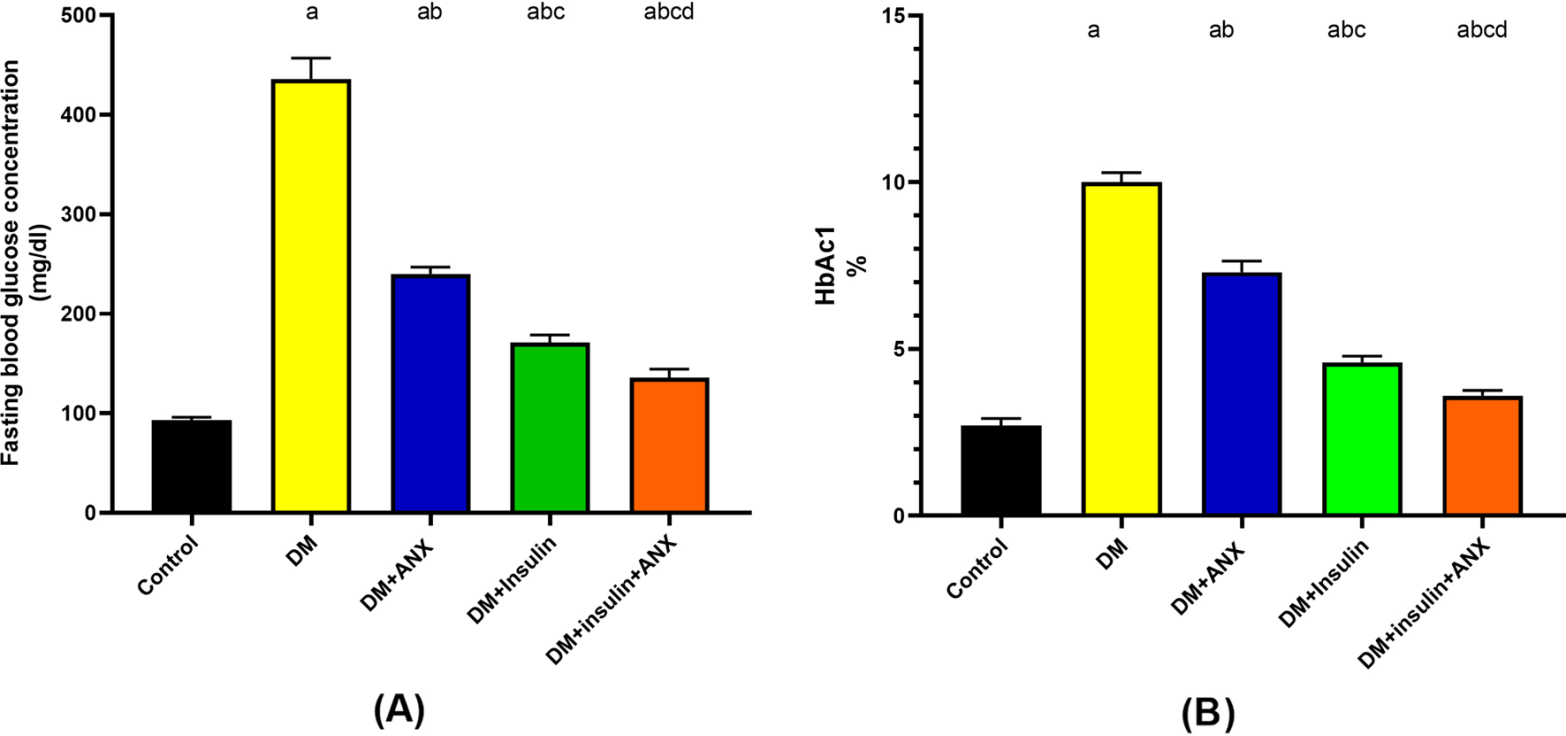
Effect of ANX plus insulin on fasting blood glucose concentration (**A**) and glycated hemoglobin (**B**), in STZ-induced diabetic rats. Data were analyzed and presented as the mean ± SE (n = 6). ^a^ *p* < 0.05 versus control group; ^b^ *p* < 0.05 versus DM group; ^c^ *p* < 0.05 versus DM + ANX group and ^d^ *p* < 0.05 versus DM + insulin group. (STZ, streptozotocin; ANX, antox; DM, diabetes mellitus and SMG, submandibular gland).

After four weeks, the DM group showed a significant elevation in both FBG concentration and HbA1c% in comparison to the other studied groups (*p* < 0.0001, each). The DM + ANX group had significantly decreased FBG and HbA1c compared to the DM group (*p* < 0.0001). On the other hand, the decrease in both FBG and HbA1c levels was more marked in the DM + insulin + ANX group (*p* < 0.0001, each) Fig. 2A and B.

#### SMG oxidative stress parameters and lipid peroxidation

**Figure 3:**
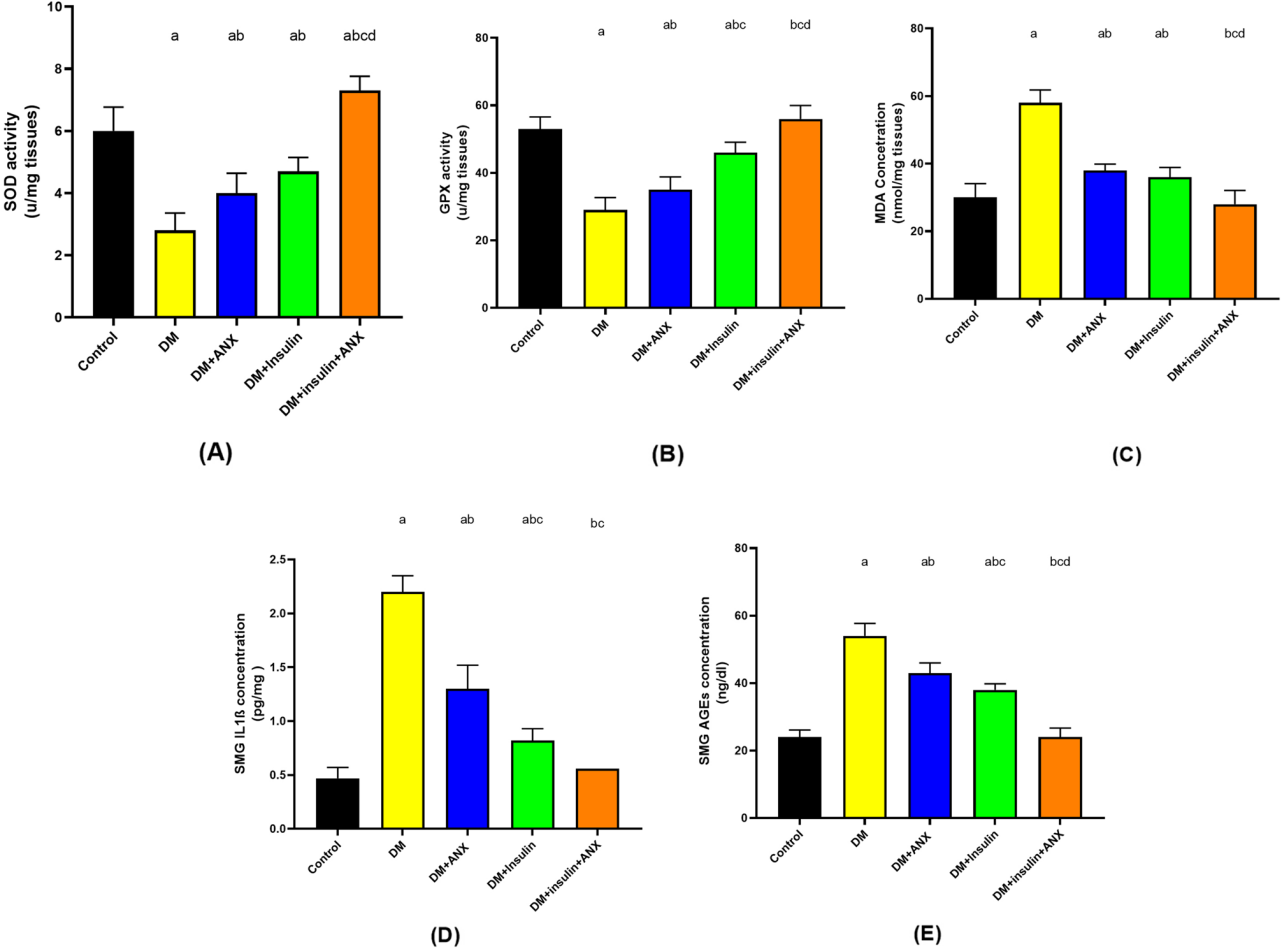
Effect of ANX and insulin on the activities of SOD (**A**), GPX (**B**) and MDA concentration (**C**) IL-1β concentration (**D**) and AGEs concentration (**E**) in SMG tissues of STZ-induced diabetic rats. Data were presented as the mean ± SE (n = 6). ^a^ *p* < 0.05 versus control group; ^b^ *p* < 0.05 versus DM group; ^c^ *p* < 0.05 versus DM + ANX group and ^d^ *p* < 0.05 versus DM + insulin group. (STZ, streptozotocin; ANX, antox; SOD, superoxide dismutase; MDA, malondiahyde; GPX, IL-1β, interleukine-1 beta: AGEs, advanced glycation end products; DM, diabetes mellitus and SMG, submandibular gland).

In comparison to the control group, the SOD activity of DM rats was considerably reduced (*p* < 0.0001). There was a significant increase in SOD activities in the DM + ANX, DM + insulin, and DM + insulin + ANX groups compared to the DM group (*p* = 0.009, *p* < 0.0001, and *p* < 0.0001, respectively) Fig. 3A.

According to the GPX activity, the DM group displayed a significant reduction when compared to the control group (*p* < 0.0001). In diabetic rats, GPX activity was elevated significantly in the DM + ANX and DM + insulin groups (*p* = 0.02 and *p* < 0.0001, respectively), although not to the same extent as in the control group (*p* < 0.0001 and *p* = 0.01, respectively). However, with the DM + insulin + ANX group, GPX activity returned to normal as in the control group (*p* = 0.5). The elevation of GPX activity was more significant in the DM + insulin + ANX group when compared with the administration of either DM + ANX or DM + insulin groups (*p* < 0.0001 and *p* = 0.0004, respectively) Fig. 3B.

As a lipid peroxidation indicator, MDA concentrations increased significantly in the DM group compared to the control group (*p* < 0.0001). With the DM + ANX and DM + insulin groups, there was a significant reduction in MDA concentrations when compared to the DM group (*p* < 0.0001, each) but they still differed from the control group (*p* = 0.001 and *p* = 0.04, respectively), while the DM + insulin + ANX group showed a return of the MDA concentration to normal as the control group (*p* = 0.9) Fig. 3C.

#### ANX with insulin displayed anti-inflammatory effects on SMG IL1β concentration in diabetic rats

In the DM group, SMG Il-1β concentration was significantly increased compared to other study groups (*p* < 0.0001, each). The DM + insulin group showed a significantly increased SMG IL1β concentration compared to the control group (*p* = 0.03), with no significant differences to the DM + insulin group or the DM + insulin + ANX group (*p* = 0.2) Fig. 3D.

#### Co-administration of ANX with insulin-dampened high AGE salivary concentration in diabetic rats

There was a significant increase (about double) in the secreted AGE concentration in the saliva of the DM group when compared to the control group (*p* < 0.0001). Administration of insulin alone or with ANX diabetic rats decreased AGE concentration significantly compared with the DM group (*p* < 0.0001 each) but did not return to normal as in the control group (*p* < 0.0001 each). However, in the combined therapy group (DM + insulin + ANX), the salivary AGE concentration was decreased to normal, compared to the control group (*p* > 0.9),) and still significantly lower compared with the DM + insulin and DM + ANX groups (*p* < 0.0001, each) Fig. 3E.

#### Co-administration of ANX with insulin returned mRNA expression of proinflammatory and fibrosis markers related genes to normal after were elevated in diabetic rats

The DM group had significantly increased proinflammatory cytokines such as IL-6, IL1β, NFkb, TNFα, and TGFβ compared with the control group. Administration of insulin alone (DM + insulin) or ANX alone (DM + ANX) in diabetic rats significantly decreased proinflammatory cytokines compared to the control group. Interestingly, the DM + insulin + ANX group significantly lowered the expression of these proinflammatory markers to normal compared to the control group Table 2.

**Table 2:** Effects of co-administration of ANX with insulin on mRNA expression of pro-inflammatory and fibrosis markers related genes in STZ-induced diabetes in rats. Data are expressed as mean ± SE and analyzed by one-way ANOVA:

	Control	DM	DM + ANX	DM + insulin	DM + insulin + ANX
IL6	1.03 ± 0.05	1.5 ± 0.15^ac^	1.4 ± 0.13^ac^	1.4 ± 0.14^ac^	1.16 ± 0.1^bd^
ILIβ	1.0 ± 0.06	1.43 ± 0.2^ac^	1.41 ± 0.1^ac^	1.38 ± 0.14^ac^	1.2 ± 0.14^bd^
NF KB	1.0 ± 0.0	1.33 ± 0.11^ac^	1.27 ± 0.13^ac^	1.25 ± 0.12^ac^	1.06 ± 0.08^bd^
TNFα	1.03 ± 0.05	1.28 ± 0.23^ac^	1.28 ± 0.11^ac^	1.3 ± 0.14^ac^	1.1 ± 0.07^bd^
TGGFβ	1.0 ± 0.09	1.42 ± 0.13^ac^	1.4 ± 0.09^ac^	1.42 ± 0.1^ac^	1.15 ± 0.1^bd^

ANX; antox, DM; diabetes mellitus, IL8; interleukin 8, ILIβ; interleukin 1 beta, NF KB; nuclear factor kappa b, TNFα; tumor necrotic factor alpha and TGFβ; transforming growth factor beta).

^a^*p* < 0.05 when compared with the control.

^b^*p* < 0.05 when compared with diabetic.

^c^*p* < 0.05 when compared with combined therapy (DM+insulin+ANX).

^d^*p* < 0.05 when compared with the group taking insulin alone (DM+insulin) OR with the group taking ANTOX alone (DM+ANX.

#### ANX plus insulin repelled the high expressions of Receptor for AGE products in diabetic rats

The expression of RAGE in the SMG of the DM group was significantly higher compared with the control group. Administration of either insulin (DM + insulin) or ANX (DM + ANX) or both (DM + insulin + ANX) has significantly decreased RAGE expressions compared to the DM group. Adding ANX to insulin (DM + insulin + ANX group) in the protocol of management significantly decreased RAGE when compared with those treated with insulin alone (DM + insulin group) (*p* ≤ 0.05).

Induction of hyperglycaemia resulted in a significant increase in PKC expression in the SMG in comparison with the control group. Administration of insulin alone (DM + insulin) reduced PKC in comparison with the DM group. However, the DM + insulin + ANX group significantly reversed the elevated PKC expression in the DM group to become as normal as the control group (*p* ≤ 0.05) Table 3.

**Table 3:** Effects of co-administration of ANX with insulin on mRNA expression of AGR/RAGE pathway in STZ-induced diabetes in rats. Data are expressed as mean ± SE:

	Control	DM	DM + ANX	DM + insulin	DM + insulin + ANX
RAGE	1 ± 0.0	6.2 ± 1.8^ac^	5.3 ± 0.12^ac^	5.5 ± 1.15^ac^	3.75 ± 1.2^bd^
PKC	1.01 ± 0.04	1.28 ± 0.19^ac^	1.26 ± 0.17^ac^	1.28 ± 0.16^ac^	1.1 ± 0.13^bd^

RAGE; receptor for advanced glycation end products and PKC; protein kinase C.

^a^*p* < 0.05 when compared with the control.

^b^*p* < 0.05 when compared with diabetic.

^c^*p* < 0.05 when compared with combined therapy (DM+insulin+ANX).

^d^*p* < 0.05 when compared with the group taking insulin alone (DM+insulin) OR with the group taking ANTOX alone (DM+ANX.

### Histological results

#### Results of H&E staining

**Figure 4:**
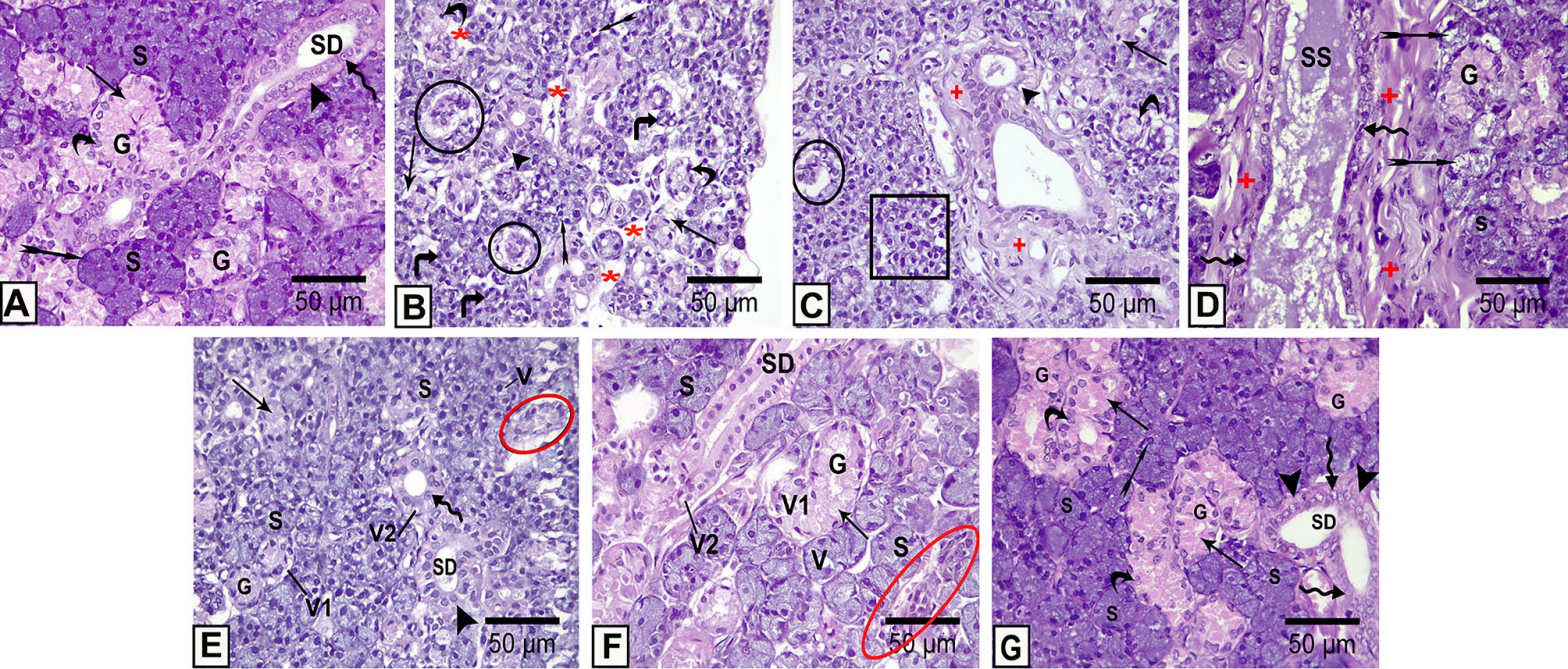
Photomicrographs of sections of the SMG from the different studied groups; **(A):** The control group shows well-defined closely packed serous acini (S), granular convoluted tubules (G), and striated ducts (SD). The acinar pyramidal cells have darkly stained cytoplasm and spherical basophilic nuclei (bifid arrow). The striated duct is lined by columnar cells having characteristic basal striations (arrowhead) and oval vesicular nuclei (zigzag arrow). The granular convoluted tubules are lined by columnar cells having abundant apical dark-stained eosinophilic granules (arrow) and basal rounded nuclei (curved arrow). (**B-D):** The DM group shows parenchymal destruction with degenerative areas (*), apparent shrunken acini (angled arrow), and the acini losing their uniform typical architecture (rectangle). The granular convoluted tubules appear atrophied leaving remnants (circle) and their cells display darkly stained nuclei (curved arrow) and cytoplasmic vacuoles (arrow). The acinar cells appear with intracytoplasmic vacuoles of different sizes (tailed arrow). Some ducts show marked dilatation with stagnant secretion (SS), flattening of its epithelial lining cells (zigzag arrow) cytoplasmic vacuoles (arrowhead), and surrounded by extensive connective tissue stroma ( +). **(E, F):** The DM + ANX/The DM + insulin groups respectively show cytoplasmic vacuoles (V, V1 &V2) in cells of serous acini(S), granular convoluted tubules (G), and striated ducts (SD) respectively. Some acini still show disturbed architecture (oval red shape). Some cells of granular convoluted tubules exhibit diminished acidophilic content (arrow). **(G):** The DM + insulin + ANX group shows more or less normal histological appearance; closely packed acini (S) having pyramidal cells with rounded nuclei (bifid arrow). The granular convoluted tubules (G) appear to have abundant eosinophilic granules (arrow) and basal nuclei (curved arrow). The striated ducts (SD) exhibit basal striation (arrowhead) with round nuclei (zigzag arrow) **(H & E, X 400).**

The control group displayed normal secretory acini and duct systems in SMG Fig. 4A.

The DM group showed obvious submandibular parenchymal destruction and altered architecture; the ducts and the secretory acini were closely packed, and degenerative areas of connective tissue stroma were obvious. There was varying intracytoplasmic vacuolisation and darkly stained nuclei in most acinar cells, cells of granular convoluted tubules, and striated ducts. There was an apparent shrinkage of several serous acini. Some granular convoluted tubules displayed varying degrees of disturbed architecture; the eosinophilic contents of secretory granules were reduced or even disappeared, and some tubules became degenerative while others atrophied, leaving only remnants. Some ducts revealed marked flattening of their epithelial lining, leading to their dilation and stagnation of secretions, and they were surrounded by extensive connective tissue stroma. The connective tissue stroma showed dilated, congested blood vessels (Fig. 4B, C, and D).

Specimens from diabetic animals treated with either ANX or insulin showed some histological improvement. Yet, most of the acinar, granular convoluted tubules, and ductal cells still have vacuoles, and some show lost and coalesced architecture (Fig. 4E,F).

Supplementation of ANX in association with insulin in diabetic rats revealed marked improvement of the glandular histological structure. The serous acini were closely packed together, and the granular convoluted tubules exhibited uniform distribution among the secretory acini. Most acini and ducts were compared to the control group Fig. 4G.

#### Results of Masson’s trichrome staining

**Figure 5:**
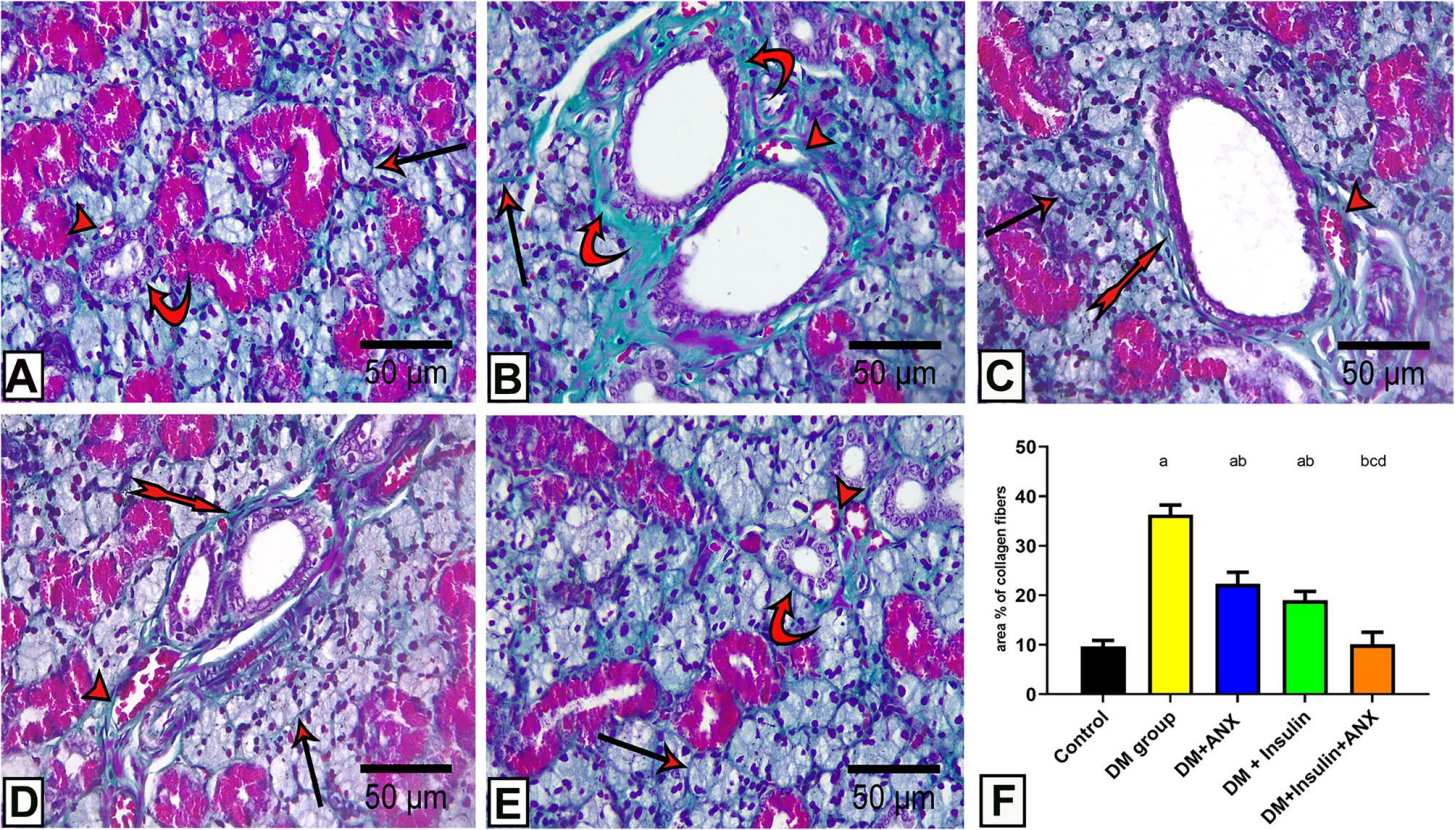
Photomicrographs of submandibular gland sections from different studied groups; **(A):** control group shows scanty collagen fibers surrounding the acini (arrow), blood vessels (arrowhead), and striated ducts (curved arrow). **(B):** The DM group showing abundant deposition of collagen fibers around acini (arrow), ducts (curved arrow) and blood vessels (arrowhead). **(C, D):** The DM + ANX/The DM + insulin groups, respectively show mild deposition of collagen fibers around striated ducts (bifid arrow) and blood vessels (arrowhead). Notice some acini are still surrounded by a considerable amount of collagen fibers (arrow) **(E):** The DM + insulin + ANX group shows very little amount of collagen fiber surrounding the acini (arrow), blood vessels (arrowhead), and striated ducts (curved arrow). **(Masson trichrome, X 400)** (**F):** Effect of ANX plus insulin on area % of collage fibers in SMG streptozotocin-induced diabetic rats. ^a^ *p* < 0.05 versus control group; ^b^ *p* < 0.05 versus DM group; ^c^ *p* < 0.05 versus DM + ANX group; and ^d^ *p* < 0.05 versus insulin-treated group (ANX, antox; DM, diabetes mellitus).

The Masson-stained sections of SMG of the control group demonstrated scanty amounts of collagen fibres that appeared as green colouration in the connective tissue surrounding the striated ducts and blood vessels (Fig. 5A). However, the DM group exhibited an abundant deposition of collagen fibres around the intralobular secretory acini and ducts in addition to that surrounding the blood vessels (Fig. 5B). Supplementation of either ANX or insulin or ANX in association with insulin ameliorated these changes (Fig. 5C,D,E). The quantitative evaluation, done by calculating the area percentage of collagen fibre deposition, proved a significant statistical reduction after administration of either ANX or insulin or ANX in association with insulin (Fig. 5F). Statistically, the DM group showed a significant elevation of the area percentage (*p* < 0.0001) compared to other studied groups. Supplementation of either ANX or insulin significantly reduced the area percentage (*p* < 0.0001), while supplementation of ANX in association with insulin showed no significant difference from the control group.

#### Results of immuno-histochemical staining of 8-OHdG

**Figure 6:**
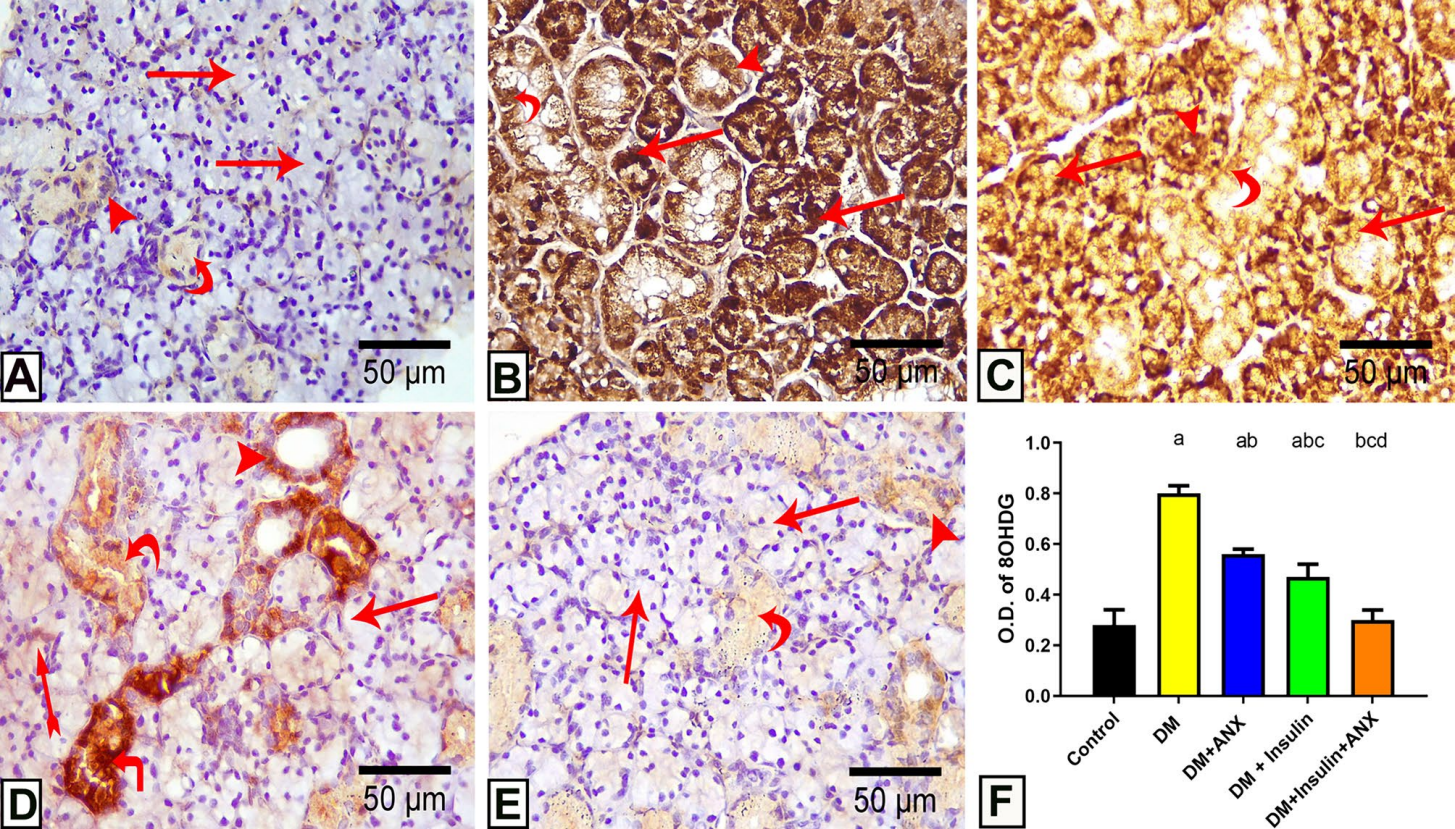
photomicrographs of submandibular gland sections from different studied groups: **(A):** control group shows Negative cytoplasmic immunoreaction for 8-OHdG in the acini (arrow), granular convoluted tubules (curved arrow), and striated ducts (arrowhead). **(B):** The DM group showing intense positive immunoreaction for 8-OHdG in cytoplasm acini (arrow), granular convoluted tubules (curved arrow), and striated ducts (arrowhead). **(C, D):** The DM + ANX/The DM + insulin groups respectively show some acini with mild reaction (arrow) and some still have an intense reaction (tailed arrow). Also, some granular convoluted tubules appear with mild reaction (curved arrow) while others with intense reaction (angled arrow). The ducts show mild reaction (arrowhead). **(E):** The DM + insulin + ANX group shows faint or nearly negative immunoreaction to 8-OHdG in the acini (arrow), granular convoluted tubules (curved arrow), and striated ducts (arrowhead) **(8-OHdG immunostaining, X 400). (F):** Effect of ANX plus insulin on OD of 8 OHdG in SMG streptozotocin-induced diabetic rats ^a^ *p* < 0.05 versus control group; ^b^ *p* < 0.05 versus DM group; ^c^ *p* < 0.05 versus DM + ANX group; and ^d^ *p* < 0.05 versus insulin-treated group (ANX, antox; DM, diabetes mellitus).

Sections from the control group showed negative cytoplasmic immunoreactions observed in the acini, granular convoluted tubules, and striated ducts of the control group (Fig. 6A).

The DM group demonstrated an intense positive immunoreaction for 8-OHdG in the cytoplasm of the acini, granular convoluted tubules, and striated ducts (Fig. 6B). In the DM + ANX and DM + insulin groups, some acini showed mild positive cytoplasmic immunoreactivity, while other acini still had an intense reaction to 8-OHdG (Fig. 6C,D). ANX administration concurrently with insulin revealed that acini, granular convoluted tubules, and intralobular striated ducts displayed a faint or nearly negative immunoreaction to 8-OHdG (Fig. 6E).

Statistical analysis of the OD of 8-OHdG immune expression ascertained a significant elevation (*p* < 0.0001) in the DM group compared to the control group. While a significant reduction (*p* = 0.0004) was noticed in the group treated with either ANX or insulin. Further reduction of 8-OHdG immune expression (*p* < 0.0001) was affirmed in the group that received both ANX and insulin (Fig. 6F).

#### ***Results of immuno-histochemical staining alpha-smooth muscle actin (α-SMA***)

**Figure 7:**
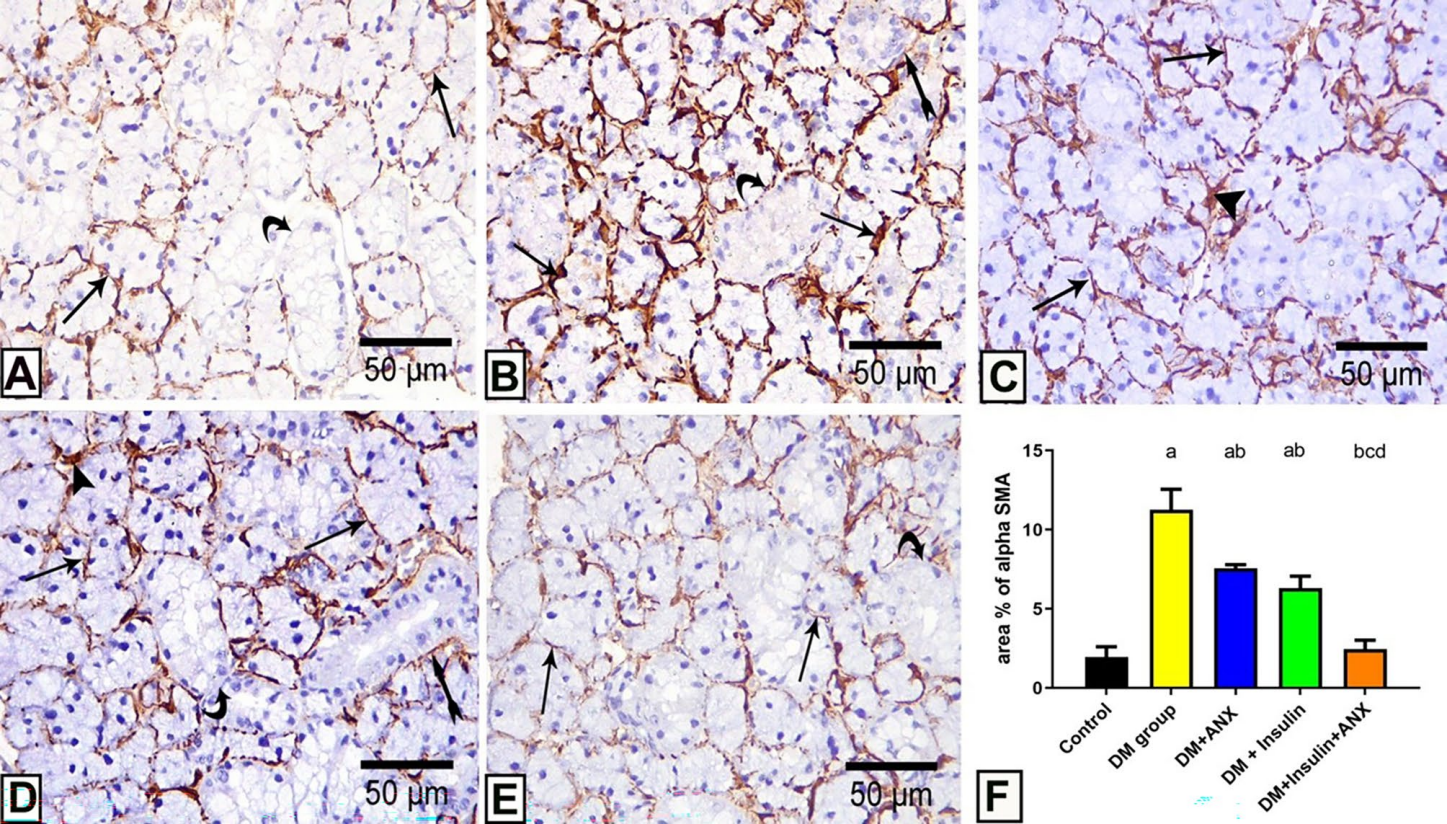
photomicrographs of submandibular gland sections from different studied groups; **(A):** control group showing faint immunoreaction for α-SMA in the myoepithelial cells surrounding the acini (arrow) and negative reaction around granular tubules (curved arrow). **(B):** The DM group showing strong positive immunoreaction at the myoepithelial cells surrounding the acini (arrow) and positive reaction around granular tubules (curved arrow) notice negative reaction around the duct (bifid arrow). **(C, D):** The DM + ANX/The DM + insulin groups respectively show some acini with a faint immune reaction to α-SMA at the myoepithelial cells surrounding the acini (arrow), around granular tubules (curved arrow) and around the duct (bifid arrow) while some acini with a strong positive reaction to α-SMA (arrowhead). **(E):** The DM + insulin + ANX group shows a faint immune reaction to α-SMA surrounding the acini (arrow) with a negative reaction at the periphery of the granular tubules (curved arrow). **(Immunostaining for α-SMA X 400) (F)** Effect of ANX and insulin on area % of α SMA in SMG streptozotocin-induced diabetic rats. ^a^ *p* < 0.05 versus control group; ^b^ *p* < 0.05 versus DM group; ^c^ *p* < 0.05 versus DM + ANX group; and ^d^ *p* < 0.05 versus insulin-treated group (ANX, antox; DM, diabetes mellitus).

Regarding the α-SMA expression in the control group, a small rim of weak positive immunoreaction to α-SMA was observed at the myoepithelial cells surrounding the acini, and the intercalated ducts, whereas no immunoreaction to α-SMA was noticed at the periphery of the granular ducts (Fig. 7A).

Concerning the DM group, a strong positive immunoreaction to α-SMA was demonstrated surrounding the acini (Fig. 7B). In the ANX-treated and insulin-treated groups, most of the acini exhibited minimal immunoreaction to α-SMA; however, a few acini appeared with strong reactions (Fig. 7C,D). Co-treatment with ANX and insulin resulted in an improvement of the immunoreaction to the α-SMA and an approximation to the control group (Fig. 7E). On statistically assessing the area percentage of α-SMA immunoreaction, the DM group showed a significant increase (*p* < 0.0001) when compared to other studied groups. Although diabetic rats treated with ANX or insulin exhibited a significant decrease, the combination of both ANX and insulin led to further improvement with no significant difference from the control group (Fig. 7F).

#### Results of immuno-histochemical staining BAX

**Figure 8:**
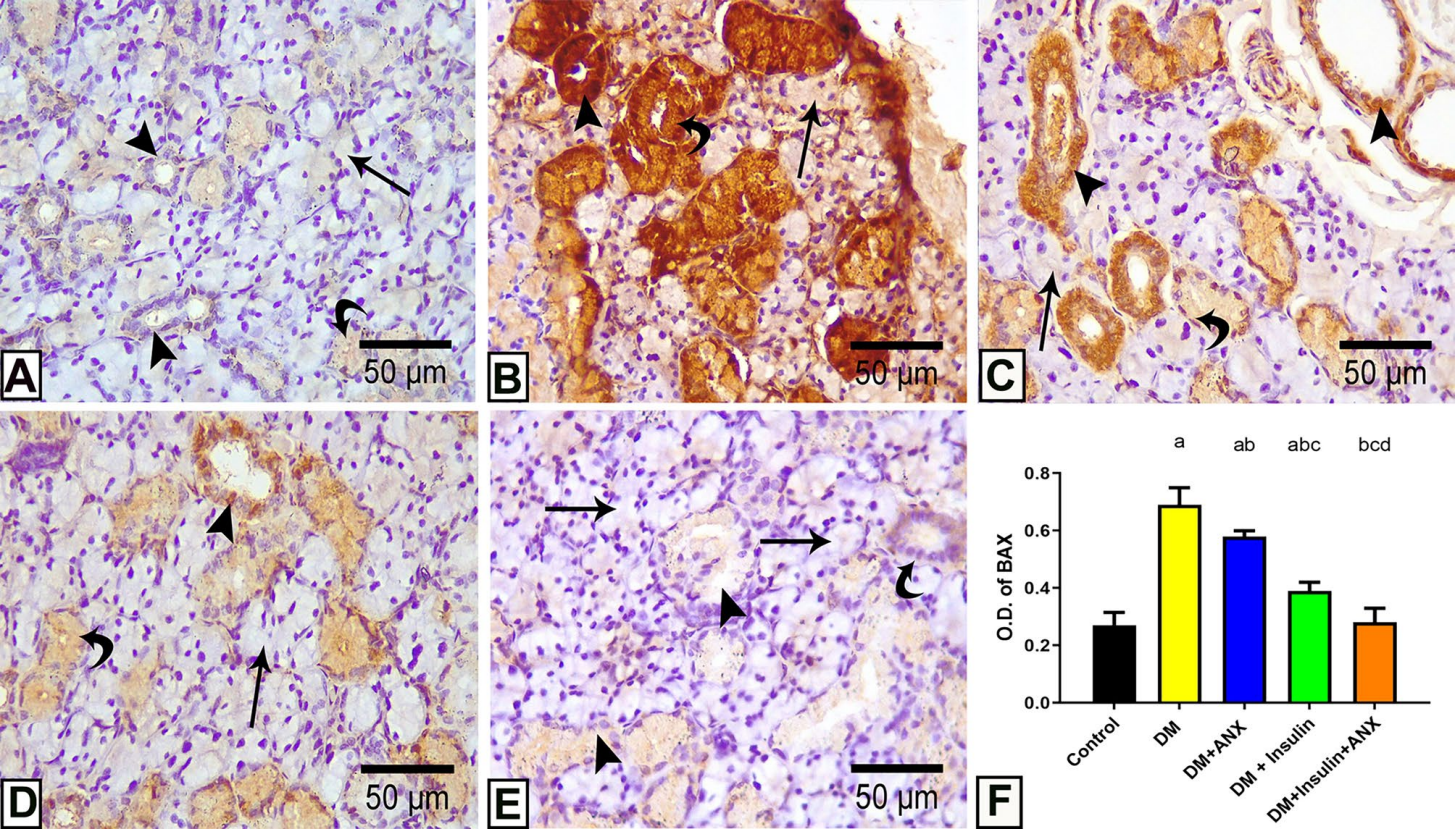
Photomicrographs of submandibular gland sections from different studied groups: **(A):** The control group shows negative cytoplasmic immunoreactivity for BAX in the acini (arrow), granular convoluted tubules (curved arrow), and striated ducts (arrowhead). **(B):** The DM group showing strong positive immunoreaction for BAX in cytoplasm acini (arrow), granular convoluted tubules (curved arrow), and striated ducts (arrowhead). **(C, D):** The DM + insulin/ANX groups showing mild reaction in the acini (arrow) and granular convoluted tubules (curved arrow) while the striated ducts show intense reaction (arrowhead). **(E):** The DM + insulin + ANX group shows faint immunoreactivity to BAX in the acini (arrow), granular convoluted tubules (arrowhead), and striated ducts (curved arrow). **(BAX immunostaining, X 400)** (**F):** Effect of ANX plus insulin on OD of BAX streptozotocin-induced diabetic rats. ^a^ *p* < 0.05 versus control group; ^b^ *p* < 0.05 versus DM group; ^c^ *p* < 0.05 versus DM + ANX group; and ^d^ *p* < 0.05 versus insulin-treated group (ANX, antox; DM, diabetes mellitus).

The control group showed negative cytoplasmic immunoreactivity to BAX in the acini, convoluted, and striated ducts (Fig. 8A). The DM group showed a massive increase in BAX immuno-reactivity in all constituents of the field Fig. 8B, while the addition of either ANX or insulin showed a mild reaction in the acini and granular convoluted tubules; furthermore, the striated ducts displayed a severe reaction (Fig. 8C,D). A very weak or even negative expression of BAX was noticed in the group concomitantly treated with both insulin and ANX (Fig. 8E).

According to the OD of BAX in the SMG section, slides from the DM group showed significant elevation (*p* < 0.0001) compared to all studied groups. ANX-treated and insulin-treated diabetic animals displayed significant elevations (*p* = 0.002 and *p* = 0.005) compared to ANX plus insulin. Co-administration of ANX with insulin showed no significant difference (*p* = 0.9) compared to the control group (Fig. 8F).

### Morphometric analysis

Regarding the acinar diameter, acinar epithelial height, and epithelial height of the striated duct of the submandibular gland, the diabetic group showed a highly significant reduction in their measurements when compared to the control group. However, the diameter of striated ducts exhibited a significant increase in the DM group when compared to the control group. The addition of ANX to insulin in the treatment of diabetic rats showed normalisation of these parameters (Table 4).

**Table 4:** Morphometric analysis of SMG acinar diameter (AD) (μm), acinar epithelial height (AEH) (μm), the diameter of striated duct (SDD) (μm), and epithelial height of striated duct (DEH) (μm), in the different studied groups via the ANOVA (analysis of variance) test. Data are represented as Mean ± SD.

Group	Acinar diameter (AD)(μm)	Acinepithelial height (AEH) (μm)	Diameter of striated duct (SDD) (μm)	Epithelial height of striated duct (DEH)(μm)
Control	31.7 ± 2.6	12 ± 3.9	39 ± 5.9	10 ± 1.5
DM	14.5 ± 2.7^a^	4.3 ± 0.9^a^	55 ± 14^a^	5.6 ± 0.7^a^
DM + ANX	22.9 ± 1.04^ab^	7 ± 0.35^a^	43 ± 1.8	7.4 ± 0.7^a^
DM + insulin	26.4 ± 4.3^ab^	9.6 ± 2.5^b^	32.3 ± 6.4^b^	9.5 ± 1.4^bc^
DM + insulin + ANX	30.8 ± 2.3^bc^	12 ± 3.5^bc^	37 ± 5.4^b^	10 ± 1.1^bc^

^a^*p* < 0.05 significant in relation to the control group,

^b^*p* < 0.05 significant in relation to DM,

^c^*p* < 0.05 significant in relation to DM+ANX,

^d^*p* < 0.05 significant in relation to DM+ ANX+ Insulin. (ANX: antox, DM: diabetes mellitus).

## Discussion

The widespread prevalence of DM in humans was the most important motivation for conducting this study, which investigated the associated molecular mechanisms brought on by AGE formation and activation of RAGEs downstream pathways to compare the effects of administration of insulin alone versus insulin plus ANX in preventing hyperglycaemic damage to SMG.

Salivary gland structural changes and dysfunction are common complications associated with diabetic patients. Per a previous study^29^, induction of diabetes in this research was performed by a single injection of STZ (50 mg/dL): an adequate dose to produce a diabetogenic effect with 100% survival in rats. This decreased the insulin plasma level and initiated insulin-dependent diabetic syndrome.

ANX (a multivitamin) was chosen in this study for its powerful antioxidant effects^31^. According to a prior study^18^; Vitamins C and E and selenium each exhibited suppressing roles against AGE production, anti-apoptosis effects, and anti-inflammatory roles.

The results of our investigation showed a marked decrease in SMG weight. According to previous research^40^, this could be due to the induction of ROS, inflammation, apoptosis, degenerative or even atrophic changes associated with hyperglycaemia, and the gland using its extracellular and intracellular components as a substitute energy source. This is also supported by the results of Stewart et al.^10^, who detected a reduction in the size of all salivary glands, including SMG, in diabetic female rats. In addition, the dysfunction of SMG in the diabetic group was detected by the reduction of the total amount of saliva, the salivary flow rate, and salivary amylase α concentrations. This aligns with Mata et al.^1^, who previously reported that DM causes impairment in saliva secretion and composition even in well-controlled human patients. These results were referred to as hyperglycaemia, as glucose is converted to sorbitol-consuming NADPH, which is necessary for the glutathione antioxidant effect, leading to oxidative stress and cell damage^33^.

In the current study, SOD and GPX activities were decreased, with an increased concentration of lipid peroxidation end product (MDA), salivary AGEs, which originally arise from non-enzymatic reactions between extracellular proteins and glucose salivary RAGEs, and proinflammatory (IL1β, IL8, TNFα, and NFκB) markers, according to a prior study^41^. The relevance of AGE-RAGE interactions and their association with hyperglycaemic harmful effects in our study are supported by previous studies carried out on insulin resistance, β cell failure, and hyperglycaemic complications. These studies considered AGEs as biomarkers and even predictors of hyperglycaemic complications. This conforms to the fact that DM induces oxidative stress.

Such outcomes were consistent with multiple experiments that focused on the altered SOD and turned on many pathways entailed in the pathogenesis of diabetic complications, excessive formation of AGEs, elevated expression of its receptors and their activating ligands^42^ and PKC isoforms^43^. According to Coughlan et al.^44^, the induction of ROS generations, such as RAGE, induces mitochondrial permeability transition, which thereafter causes NADH-dependent increased production of mitochondrial superoxide radicals. These lead to the activation of proinflammatory pathways, resulting in long-lasting epigenetic changes that lead to sustained expression of proinflammatory genes even after reaching the normoglycemic state (‘hyperglycaemic memory’)^45^.

Giacco and Stewart et al.^10,45^ explained the AGE-induced cell damage by many mechanisms such as the alternation function of a protein that is modified by AGEs, abnormal interaction of extracellular matrix components and matrix receptors (integrin) with AGEs, and finally, activation of RAGEs by AGEs in cells such as endothelial and smooth muscle cells of the blood vessels, causing ROS generation, activation of the transcription factor NF-kB, and subsequent alternation of gene expressions. Also, induction of cell death by activation of the apoptotic pathway occurs through a novel pathway, as RAGE activation leads to rupture of IL-8 vesicles. The degranulated IL-8 acts through its receptors, IL-8Rs, to increase the activity of caspases through FasL activation^8^.

Our study revealed that ANX co-supplementation with insulin averts oxidative stress and inflammatory processes induced by hyperglycaemia. This was in harmony with Daoud et al.^46^, who stated that ANX inhibited free radical generation and decreased inflammatory reactions in a rat model of trichinosis. Another study showed that ANX modifies the testicular injury caused by cadmium in adult male albino rats by inducing antioxidant effects with suppression of caspase levels^47^. It concluded that treatment with ANX twice daily with metformin for three months is a useful therapy in type 2 diabetic patients as it is well tolerated and devoid of any side effects. Even so, it led to improvement and caused a decrease in FBG, HbA1c, TC, TG, and LDL and a significant increase in HDL concentrations^48^.

The current work demonstrated that supplementation of ANX with insulin in diabetic rats normalised the high levels of salivary AGEs and SMG expression of RAGE, which was partially decreased with insulin treatment alone. In agreement with these results, various studies used dietary supplements to ameliorate the increased expression of the AGE/RAGE axis, as with a prior study in which glutamine (Gln) and inosine were administered respectively^49,50^.

In a recent study, the high concentration of AGEs in the diabetic group had significantly upregulated submandibular gland PKC and TGFβ. This is in line with several previous studies, such as Ganesan et al.^51^, who reported glucose-induced translocation of protein kinase C in diabetic rat pancreatic islets, Lindschau et al.^52^ who related the diabetic-increased expression of glucose-induced TGF-β and TGF- β -R1 in lymphocytes to PKC-α, and Giacco^53^, who stated that AGEs in the mesangial cells of neonates promote oxidative stress and actuate PKC-beta (II). Even more, many studies pointed out that hyperglycaemia associated with diabetic rats may indirectly actuate PKC isoforms through ligation of the RAGE and increased activity of the polyol pathway^47,48^.

In their study, Hsieh et al.^54^ noted that hyperglycaemia-activated PKC leads to the induction of NF-κB through the HMGB1/RAGE axis, which results in the subsequent exacerbation of inflammation via the production of oxidative stress. Also, some studies revealed that lipid droplet deposition in epithelial cells in salivary glands with excessive free fatty acid-induced lipotoxicity and cell apoptosis induces an inflammatory response in salivary glands^55,56^. On the other hand, Eweda et al.^57^ referred to these previous hyperglycaemia-induced insults to the development of polyuria and osmotic diuresis that caused dehydration and hyposalivation.

A previous study with diabetic rats^57^ that used bitter gourd, a powerful nutrient-dense fruit extensively rich in antioxidant vitamins recorded stimulation of high levels of insulin and auto-phosphorylated insulin receptors, resulting in enhancement of glucose uptake and use. These results may explain our findings, as the treatment with ANX and insulin significantly reversed PKC expression and TGF-β to a nearly normal level compared to both the diabetic and insulin-treated groups, thus suggesting that ANX may increase the sensitivity of insulin to bind to its receptor, confirming our hypothesis of the underlying mechanism of the anti-diabetic action of ANX.

From a histological point of view, the recent study revealed histological changes in the SMG of diabetic rats, varying among atrophy, shrinkage, extensive fibrous tissue infiltration, nuclear changes, and the cytoplasmic vacuolation of acinar cells. Ship and Fischer^58^ attributed the cytoplasmic vacuolations to fatty degeneration and cytoplasmic deposition of degenerative products, while the reduction in acinar size and gland atrophy in diabetic rats was attributed to fibrous or fatty infiltration in addition to the affection of the cell membrane integrity and permeability, resulting in changes in the haemostasis of the cell volume^59^. Therefore, these severe structural changes are the direct cause of the reduction in SMG weight and the functional changes in the form of xerostomia (hyposalivation).

In contrast, many studies^60,61^ explained these functional changes based on altered intracellular signals induced by DM-associated oxidative stress and lipid peroxidation. Li et al.^62^ considered it the key factor that stimulated α-collagen expression with excess collagen synthesis. Another previous study^63^ provided compelling evidence that DM triggers apoptosis through increased expression of BAX in the diabetic group, which is similar to our findings. These altered intracellular signals in turn attack the chromatin, causing DNA fragmentation and ending in cell death^1^.

8-OHdG was first discovered as one of more than 20 oxidative bases. It is a sensitive parameter for DNA damage and the most studied base damage product^64,65^. In the current study, the fact that the diabetic rats had higher salivary 8-OHdG levels than the healthy control group lends more credence to the theory that DM is linked to elevated oxidative stress.

Notably, our study reported statistically significant dilated excretory ducts, reduced epithelial height of striated ducts with retained secretion, and even degenerated epithelial lining in the SMG of diabetic rats. These findings were in line with earlier studies^66,67^ that excluded the intercalated duct epithelial cells from such changes and attributed these changes to the increase in the proliferative activity of the duct system.

In our study, ANX co-administration with insulin improved the histological architecture of SMG more effectively than using insulin therapy alone, and this proved statistically significant toward normalisation. The benefits of daily consumption of dietary supplements were proven by previous studies such as Anderson et al.^66^, who focused on Vitamin A supplementation, Elabasiry et al.^68^, who investigated the use of supplementary Vitamin C in type 2 diabetes, and Omar et al.^69^, who studied the effect of oral supplementation of Vitamin E.

The detected histological improvement in this study is also supported by the results of existing research^70^ stating that myoepithelial cells, intercalated ductal cells, and acinar cells have low baseline proliferation (physiological regeneration) under normal circumstances. However, when glandular injury occurs, the proliferation index increases, especially in myoepithelial cells. A prior study^71^ referred to myoepithelial proliferation as the need to compensate for the degeneration process and suggested that myoepithelial cells could act as stem or phagocytic cells and preserve the secretory functions of the gland. This explains our findings about the significantly higher expression of the α-SMA immunoreaction in the diabetic group, while this level was noticeably ameliorated after co-administration of insulin and ANX. On the other hand, this contradicts the previous conclusion of Mata^1^ concerning the inability of the gland to regenerate.

Finally, Morsy et al. and Hassan et al.^11,15^ reported that histological and immunohistological studies included that ANX supplementation reversed any testicular abnormalities (functions and histological) in diabetic male rats and parotid gland insults associated with type 1 DM experimentally induced in male rats.

## Conclusions

In conclusion, co-supplementation of ANX and insulin in diabetic rats improved SMG structural alternation and dysfunction statistically through antioxidant, anti-inflammatory, and anti-apoptotic effects. This is attributed to decreased AGE production and turn-off downstream pathways associated with RAGE activation due to the induction of diabetes.

## Data Availability

Data will be made available on request and requested from corresponding author H.M.A. Email: mmabdelkarim@zu.edu.eg.
